# Nurses’ perspectives on remote healthcare delivery and its implications for practice and education: qualitative study

**DOI:** 10.1186/s12912-026-04497-z

**Published:** 2026-03-03

**Authors:** Sıdıka Kestel, Fatos Korkmaz

**Affiliations:** Fundamentals of Nursing Department, Faculty of Nursing, Hacettepe University, Adnan Saygun Cad. D Bloklari Samanpazari Altindag, Ankara, 06200 Turkey

**Keywords:** Remote healthcare, Telehealth, Digital health, Nursing education, Telenursing, Professional competence

## Abstract

**Aim:**

To explore nurses’ perspectives on remote healthcare delivery, focusing on perceived opportunities, challenges, and implications for nursing practice and education.

**Background:**

The rapid digital transformation of healthcare has expanded the use of remote healthcare systems, reshaping how nurses deliver, coordinate, and document care. As key providers of patient-centered care, nurses play a critical role in the integration of telehealth services. Understanding their experiences is essential to inform educational preparation, workforce planning, and policy development for sustainable digital health practice.

**Design:**

A descriptive qualitative design was adopted.

**Methods:**

Semi-structured interviews were conducted with 23 registered nurses working in diverse clinical and community settings across Turkiye between February and July 2025. Data were collected via online video interviews and analyzed inductively using Braun and Clarke’s six-phase approach to thematic analysis. Reporting followed the COREQ checklist.

**Results:**

Six main themes were identified: *Perceived benefits for patients and health systems; Professional opportunities and motivation; Technological and organisational requirements; Legal*,* ethical*,* and privacy concerns; Barriers related to users;* and *Clinical appropriateness and boundaries.* Nurses emphasized that remote healthcare improves continuity of care, supports early intervention, and strengthens chronic disease management. However, limited infrastructure, workload concerns, ethical uncertainties, and digital literacy gaps remain significant challenges. The need for structured training, clear accountability, and equitable access was consistently highlighted.

**Conclusion:**

Nurses view remote healthcare as a transformative approach that enhances patient outcomes and extends professional roles beyond traditional hospital boundaries. Yet, its effectiveness depends on robust digital systems, comprehensive education, and policy frameworks that safeguard both patients and professionals.

**Clinical Trial Number:**

Not applicable. This research is a qualitative study.

## Introduction

The rapid expansion of telehealth and remote healthcare services is transforming how nurses deliver, coordinate, and document care. Remote healthcare delivery, often referred to as *telehealth* or *telemedicine*, involves using telecommunication technologies to provide health services at a distance [1]. Its applications range from real-time video consultations to remote patient monitoring and mobile health applications [2]. Advances in digital technology, the growing prevalence of chronic illness, and lessons learned during the COVID-19 pandemic have collectively accelerated the adoption of virtual health services worldwide [3]. These shifts also demand that nurses continuously strengthen their digital competencies, communication skills, and ethical awareness to ensure safe and effective care delivery in technology-enabled environments.

Remote healthcare enables continuous monitoring of patients’ symptoms, treatment adherence, and physiological parameters outside the hospital, providing opportunities for early detection of complications and improved continuity of care [4, 5]. As reported by Tan, Sumner, and Wang (2024), remote systems support real-time data collection in home settings, enhancing clinical decision-making and enabling timely interventions particularly for individuals with complex or chronic conditions [6]. Similarly, Wartenberg, Elden, and Frerichs (2025) found that remote healthcare can reduce readmissions and optimize healthcare costs, reinforcing its potential for efficiency and sustainability [7].

Beyond the clinical benefits, remote healthcare expands the professional scope of nursing. Nurse-led virtual interventions have been shown to improve symptom management, treatment adherence, and patient satisfaction [8, 9]. Evidence also demonstrates the value of remote nurse monitoring in chronic disease management, including blood pressure control [10, 11] and telerehabilitation [12]. These findings position nurses not only as users of technology but as key coordinators of patient care within digital health ecosystems. This evolving role requires nurses to acquire new digital, ethical, and communication competencies areas that must be reinforced through both pre-licensure and continuing education [13].

However, the integration of remote healthcare into everyday nursing practice presents ongoing challenges. Implementation requires robust technological infrastructure, clear regulations, and well-defined professional roles. Legal and ethical issues such as data security, patient privacy, and liability remain major concerns [14, 15]. Additionally, the effectiveness of remote care depends heavily on nurses’ digital competence, confidence, and communication skills [5, 16]. Many nurses express uncertainty about how virtual interactions affect empathy, trust, and the therapeutic relationship core elements of nursing care [17].

In this context, nurses’ experiences offer vital insight into how remote healthcare can be effectively implemented and sustained. Their perspectives are essential for guiding educational strategies, shaping policy frameworks, and informing workforce development. Understanding these perspectives is particularly relevant in Türkiye, where telehealth adoption is increasing but remains uneven across regions and healthcare institutions. A deeper understanding of nurses’ perceptions can inform curricular design, professional training, and institutional strategies that prepare the nursing workforce for digitally integrated care.

Therefore, this study aimed to explore nurses’ perspectives on remote healthcare delivery, emphasizing perceived opportunities, challenges, and implications for nursing practice and education.

## Methods

### Study design

This study employed a descriptive qualitative design to explore nurses’ perspectives on remote healthcare delivery. The qualitative approach was selected to gain an in-depth understanding of nurses’ perceptions, experiences, and professional reflections on the integration of remote healthcare practices into clinical and community settings. The study was guided by a constructivist paradigm, recognizing that knowledge is co-constructed through participants’ experiences and researcher interpretation. To ensure comprehensive and transparent reporting, the study followed the Consolidated Criteria for Reporting Qualitative Research (COREQ) checklist [18].

### Participants and setting

Participants were selected through purposive, maximum variation, and snowball sampling strategies to capture diverse experiences across different clinical contexts. Inclusion criteria required at least one year of professional nursing experience, with participants’ experience ranging from 1 to 28 years, in hospital or community-based care.

Participants were recruited from healthcare institutions that differed in structure, function, and level of care to ensure contextual diversity. The sample included nurses working in tertiary Research and Training Hospitals and University Hospitals, secondary Public City Hospitals, and primary healthcare settings such as Family Health Centers.

In the analysis, nurses were not treated as a single homogeneous group. Work arrangements were acknowledged to differ between primary and tertiary settings, and analysis was conducted accordingly. During coding, the institution and role of each participant were noted. Themes were first compared within the same type of institution (e.g., primary care only) and then across institutions. Care was taken not to present workflows specific to a particular setting as universally applicable findings. When interpreting shared themes, the level of care (primary-tertiary) was considered.

Initial participants were contacted by one of the researchers (SK) via WhatsApp messaging and received a brief description of the study purpose and participation requirements. Following verbal agreement, a digital information sheet and consent form were provided. At the end of each interview, participants were invited to recommend colleagues from other institutions or specialties who might contribute additional perspectives.

Recruitment continued until data saturation was achieved, defined as the point at which no new codes or ideas emerged during analysis. In total, 23 registered nurses participated, representing a broad range of specialties including intensive care, internal medicine, pediatrics, cardiology, general surgery, gynecology, oncology, psychiatry, sleep medicine, pain management, community health, diabetes education, and stoma care. This variation enhanced the contextual breadth of the findings.

Although most participants were clinical nurses working in hospitals, community-based nurses from family health centers were also included. These centers represent primary care within Turkey’s healthcare system, and their inclusion allowed the study to capture differences in remote healthcare practices across care levels. Telehealth services in community settings remain relatively recent and less structurally embedded than in hospital contexts, where workflows, digital literacy, and engagement patterns may differ.

### Data collection

A semi-structured interview guide was developed after reviewing current literature [19, 20] and drawing on the research team’s expertise in nursing informatics and remote care. The guide contained nineteen open-ended questions covering demographic characteristics, institutional systems, patient profiles, and perceptions of remote healthcare (see Table 1).

**Table 1:** Semi-structured interview guide exploring nurses’ perspectives on remote healthcare delivery

Domain	Focus Area	Example Questions
Demographic and Professional Background	Personal and professional characteristics	• Can you describe your current position, clinical unit, and years of nursing experience? • What is your main area of responsibility in patient care?
Institutional System Context	Hospital information systems and documentation practices	• What hospital information system is used in your institution? •Does the system include the data nurses collect, such as care forms or follow-up records? • How would you evaluate the scope and adequacy of this content for nursing care? • How ready do you think your institution is for implementing remote healthcare?
Patient Profile and Follow-up Practices	Post-discharge needs and monitoring requirements	• How would you describe the patient population in your unit? • Which patient groups require follow-up after discharge, and why? • How is post-discharge monitoring currently performed?
Experience with Remote Healthcare	Awareness and perceptions of remote or digital monitoring	• Is there a remote monitoring system for patients in your institution? • What are your thoughts about systems designed for remote health monitoring (e.g., symptom tracking, medication management)? •What benefits or risks do you foresee for patients and for healthcare staff? • What challenges or ethical concerns might nurses face when using such systems?
Professional Roles and Competency Needs	Nursing roles, responsibilities, and required skills	• How could nurses be involved in delivering or coordinating remote healthcare services? • What competencies or training do you think nurses need to provide remote healthcare effectively? • In your opinion, what aspects of nursing practice would need to be strengthened for telehealth?
Motivation and Future Perspectives	Attitudes and willingness to work in remote healthcare	• Would you consider working in an organization that provides remote healthcare services? Why or why not? • What factors would motivate or discourage nurses from participating in such services?

A pilot study with three nurses was conducted on February 17, 2025, to ensure clarity and relevance. As no revisions were required, these data were included in the final analysis.

Given the participants’ variable shifts and geographical dispersion, all interviews were conducted online via Zoom or WhatsApp video calls, ensuring flexibility and participant comfort. Interviews were conducted by the first author (SK), an experienced qualitative researcher in telehealth. The second author, an associate professor specializing in digital health systems, provided methodological oversight.

Interviews lasted approximately 30–40 min and were audio-recorded with consent. Written informed consent was obtained electronically through signed forms returned via WhatsApp. All interviews were conducted in Turkish and later translated into English for reporting.

Recordings were transcribed verbatim and anonymized using participant codes (e.g., N1, N2). Participants reviewed their transcripts for accuracy, and no modifications were requested. All files were stored on password-protected devices accessible only to the first author. To enhance reflexivity, the interviewer maintained reflective notes after each interview, documenting assumptions, impressions, and analytic decisions throughout the process.

### Data analysis

Data were analyzed following Braun and Clarke’s (2006) six-phase thematic analysis framework [21]. Transcripts and notes were organized in MAXQDA20 software to support systematic coding.

Both researchers independently read transcripts multiple times to become familiar with the data and generate initial codes. Coding was conducted inductively, emphasizing recurring ideas and meanings in nurses’ accounts. Codes were then compared and refined through iterative team discussions until consensus was reached.

Codes were clustered into categories and emerging themes, supported by illustrative quotations. Thematic refinement continued until no new subthemes appeared, ensuring analytic saturation. To strengthen dependability, detailed analytic memos and an audit trail of all coding decisions were maintained. The final thematic framework was developed collaboratively and verified against original transcripts to ensure alignment with participants’ narratives.

### Rigor and trustworthiness

To ensure methodological rigor and trustworthiness, the study adhered to Lincoln and Guba’s (1985) criteria: credibility, dependability, confirmability, and transferability [22].

- Credibility was achieved through independent coding by both researchers and participant transcript verification. The researchers met weekly during analysis to compare codes, discuss interpretations, and reach consensus. Peer debriefing with two qualitative research colleagues further supported the accuracy of interpretations.
- Dependability was maintained by consistently applying Braun and Clarke’s (2006) six-phase framework and documenting analytic decisions in an audit trail that included memos, coding summaries, and reflective notes.
- Confirmability was strengthened through reflexive journaling. The first author kept a diary after each interview and during coding to record assumptions, reflections, and methodological decisions, ensuring findings were grounded in participants’ data rather than researcher bias.
- Transferability was enhanced by recruiting 23 nurses from 13 hospitals and community health centers across varied specialties and experience levels, providing rich contextual diversity for interpretation.

These strategies collectively enhanced transparency and confirm the credibility and trustworthiness of the findings [23].

### Ethical considerations

Ethical approval was obtained from the Social Sciences and Humanities Research Ethics Board of Hacettepe University (Approval Date: December 10, 2024; Protocol ID: 00003937850).

All participants were informed of the study objectives, procedures, and their right to withdraw at any time without consequence. Verbal and written informed consent were obtained before each interview. Confidentiality and anonymity were maintained through coded identifiers and secure digital storage. Only the first author had access to encrypted data files.

All study procedures followed the principles of the Declaration of Helsinki and institutional ethical standards for research involving human participants.

## Results

### Participants characteristics

The study included 23 registered nurses who met the inclusion criteria. Among them, three were community health nurses and twenty were clinical nurses representing a range of specialties across public hospitals and outpatient clinics, including intensive care, internal medicine, pediatrics, cardiology, general surgery, gynecology, oncology, psychiatry, pain management, diabetes education, sleep medicine, and stoma/colostomy care. Participants had a mean age of 37.9 years (SD = 12.1) and an average of 15.4 years of professional experience. Five were male and the remaining eighteen were female. All participants held a bachelor’s degree in nursing.

### Institutional context

The participants worked in institutions that used either Sisoft or Nucleus hospital information management systems. These platforms were described as primarily physician-centered, focusing on patient demographic information, medical diagnoses, and medication orders. While measurable nursing data such as vital signs and pressure injury stages were integrated into the system, documentation of nursing care processes remained largely paper-based. Several participants emphasized that these systems were inadequate in capturing the full scope of nursing practice, which often required manual recording to ensure continuity of care.

### Themes

The analysis revealed six main themes and fourteen subthemes that reflected nurses’ experiences and views regarding remote healthcare delivery:

1. Perceived Benefits for Patients and the Health System.
2. Professional Opportunities and Motivation.
3. Technological and Organisational Requirements.
4. Legal, Ethical, and Privacy Concerns.
5. Barriers Related to Users.
6. Clinical Appropriateness and Boundaries.

Each theme reflects nurses’ nuanced perspectives, revealing both optimism regarding the potential of remote healthcare and caution about its practical limitations. Rather than simply describing experiences, the thematic structure illustrates how nurses interpret and negotiate remote healthcare within existing organisational realities. Table 2 presents the themes, subthemes, and representative quotations.

**Table 2:** Themes and subthemes examples

Theme	Sub-themes	Codes	Example Quotes
1. Perceived Benefits for Patients and the Health System	1.1 Continuity of care and early intervention	• Post-discharge follow-up • Preventing readmissions • Early detection of complications	“Such a system would reduce hospitalization. For example we could intervene before a pressure ulcer becomes advanced.” (N13)
	1.2 Cost and time savings	• Reduced travel and expenses • Decreased hospital-acquired infections	“Parents could be guided remotely for fever follow-up, guiding parents remotely for fever follow-up helped minimize children’s exposure to hospital-acquired infections.” (N7)
	1.3 Support for chronic disease management	• Monitoring diabetes, hypertension, heart failure • Long-term lifestyle counselling	“…such as diet and exercise advice, patients could be followed consistently with remote sessions.” (N15)
	1.4 Improved patient self-management	• Increased treatment adherence • Greater patient responsibility	“In my view, conducting remote follow-ups helps patients stick more consistently to their medications and treatment plan.” (N8)
2. Professional Opportunities and Motivation	2.1 Expanded nursing roles and professional development 2.2 Motivation and new possibilities for younger nurses	• Greater autonomy • Care coordination	“A nurse is the one who observes and knows the patient best… therefore, we should definitely be included in telehealth services.” (N3)
		• Excitement among younger nurses • Flexible work options	“As a young nurse, I find these applications exciting… I could track how my ICU patient manages care at home.” (N1)
3. Technological and Organisational Requirements	3.1 Infrastructure and devices	• Reliable internet • Monitoring tools (BP cuff, thermometer, etc.)	“Our country is not ready in terms of infrastructure. System crashes and internet access are major issues.” (N1)
	3.2 Staffing and workflow coordination	• Clear scheduling • 24/7 availability • Defined nurse–patient matching	“There must be a clear work schedule; who the patient contacts outside office hours must be defined.” (N11)
4. Legal, Ethical, and Privacy Concerns	4.1 Data protection and confidentiality	• Secure documentation • Risk of unauthorized access	“What if someone else is listening on the patient’s or professional’s side?” (N6)
	4.2 Responsibility and accountability	• Unclear liability for adverse events • Need for national policy	“Who will take responsibility if a patient experiences complications after remote advice?” (N4)
5. Barriers Related to Users	5.1 Patient-releted barriers	• Low digital literacy • Preference for face-to-face contact	“Older patients may have difficulty or may not prefer it.” (N15)
	5.2 Loss of human connection	•Limited technology skills • Perceived extra workload .Less emotional contact with patients .Non-verbal communication limited . Nurse–patient relationship lacking closeness	“Even some experienced nurses struggle with smartphones and may see this as extra workload.” (N17). “It is difficult to understand patients’ feelings or concerns beyond what they say verbally if there is no face-to-face interaction” (N14). “The act of touching, the sound of voices and the presence of people are all part and component of nursing care. It is difficult to convey these through a screen”. (N9)
6. Clinical Appropriateness and Boundaries	6.1 *Appropriateness of patient and condition characteristics* 6.2 Appropriateness of service type	.Chronic diseases wound/ostomy care • Medication adherence • Post-operative follow-up	“…beneficial for ostomy patients complications can be managed before they become severe.” (N4)
		• Acute psychiatric crises • End-stage palliative care • Situations needing physical examination	“Not suitable patients who are closed to communication.” (N1) “Physical examination cannot be replaced, especially for young children.” (N5)

Differences across institutional settings and nursing roles were explicitly examined during analysis, enabling the identification of context-specific nuances in experiences and responsibilities. For example, primary care nurses emphasized patient education and continuity of care, whereas tertiary-level nurses highlighted complex clinical coordination and interprofessional collaboration. These contextual distinctions shaped how participants evaluated the feasibility, risks, and boundaries of remote healthcare in practice, thereby strengthening the interpretive depth of the thematic findings.

### Theme 1: Perceived benefits for patients and health systems

Nurses consistently described remote healthcare as a valuable innovation for enhancing patient outcomes, continuity of care, and system efficiency. Their reflections clustered around four subthemes:

1. *Continuity Of Care And Early Intervention,*
2. *Cost And Time Savings,*
3. *Support For Chronic Disease Management,*
4. *Improved Patient Self-Management.*

#### Continuity of care and early intervention

Participants emphasized that remote healthcare enables uninterrupted patient follow-up, allowing earlier identification and management of potential complications. Regular assessment of medication adherence, vital signs, wound healing, and other indicators in the home environment was viewed as instrumental in preventing deterioration and avoidable hospital readmissions.

I believe the system will offer many advantages, including facilitating patient follow-up after discharge, ensuring continuity of care, and intervening early in complications. For example, physical assessments such as monitoring patient compliance with medications and treatments, edema monitoring, wound care monitoring, blood pressure monitoring, and blood sugar monitoring can be performed without the patient being admitted to the hospital. (N1).

Several nurses noted that home-based assessments may yield more accurate data by reflecting patients’ natural living conditions, leading to better diagnostic precision.

In some cases, home-based monitoring may enhance diagnostic accuracy. For instance, patients are generally observed for a single night in a sleep clinic, where optimal sleep hygiene is frequently not attained. The assessment in the home environment can support proper sleep hygiene and enable the collection of more reliable data, thereby facilitating a more precise diagnosis. (N4).

This theme reflected a strong belief that remote follow-up helps maintain patient engagement and strengthens continuity of care, particularly for individuals facing mobility challenges or geographic barriers to accessing services.

#### Cost and time savings

Nurses described remote healthcare as a strategy that can reduce unnecessary hospital visits and lower costs for both patients and health systems. Participants mentioned that remote consultations save travel time and expenses and minimize exposure to hospital-acquired infections, especially among vulnerable populations such as children or immunocompromised patients.

Parents could be guided remotely for fever follow-up. Guiding parents remotely for fever follow-up helped minimize children’s exposure to hospital-acquired infections. (N7).

Remote healthcare can eliminate the need for long-distance travel for patients in rural or underserved areas, reducing the time and costs associated with accessing healthcare. (N13).

This subtheme highlights nurses’ awareness of how remote healthcare contributes to cost-effectiveness, workforce efficiency, and overall system sustainability elements that align with global digital health priorities.

#### Support for chronic disease management

Nurses widely recognized the value of remote monitoring for long-term management of chronic conditions such as diabetes, hypertension, heart failure, and respiratory diseases. They associated these systems with improved adherence to treatment, better symptom control, and opportunities for early intervention.

Many patients with diabetes or hypertension struggle to change their habits. Remote monitoring allows us to review blood sugar or blood pressure records and intervene early, before complications arise. (N6).

For heart failure patients, weight tracking and daily symptom reporting via the app can allow us to quickly detect fluid retention and adjust treatment without needing to go to the hospital. (N12).

Some nurses also highlighted that remote platforms make it easier to involve family members in care and strengthen education on medication adherence and lifestyle modification.

It can provide the opportunity to collect data by meeting with all family members simultaneously without making a home visit. (N17).

This perspective aligns with prior evidence indicating that nurse-led virtual interventions can support sustained self-care and improved health outcomes for chronic disease populations.

#### Improved patient self-management

Nurses reported that remote healthcare encourages patients to take greater responsibility for their health, enhancing treatment adherence and self-awareness.

When patients know that we monitor their blood pressure or glucose levels daily, they take more responsibility for their own health, which I believe increases their adherence to treatment. (N8).

With this system, we include patients in monitoring their symptoms, so they become more actively involved in the treatment process. In addition, we also include their family members or caregivers in the treatment process. (N15).

These statements reflect nurses’ perception that digital health systems not only extend care beyond hospital walls but also empower patients to become active participants in maintaining their well-being.

### Theme 2: Professional opportunities and motivation

Beyond improving patient outcomes, nurses viewed remote healthcare as an opportunity to redefine and expand their professional roles. They associated telehealth systems with increased autonomy, interdisciplinary collaboration, and new career possibilities. Two interrelated subthemes were identified:

1. *Expanded Nursing Roles and Professional Development,*
2. *Motivation and New Possibilities for Younger Nurses. *

#### Expanded nursing roles and professional development

Nurses emphasized that remote healthcare offers a path to broaden the scope of nursing practice beyond traditional hospital-based settings. They highlighted how digital platforms could strengthen their role in coordinating care, supporting patient education, and ensuring continuity across transitions of care. Several participants perceived this transformation as both empowering and necessary for the future of the profession.

A nurse is the one who observes and knows the patient best and knows what will be in the patient’s best interest. Therefore, we should definitely be included in remote healthcare services, and it would be wonderful. (N3).

From our profession’s perspective, someone must coordinate this process. New roles or titles such as remote care nurse coordinator, digital nurse, or telehealth nurse may emerge. (N11).

These statements illustrate how nurses see digital care models as an opportunity to assume greater responsibility, exercise professional judgment, and engage in complex decision-making processes. Participants also described remote healthcare as an environment that fosters interprofessional collaboration, particularly for chronic disease management and rehabilitation.

It provides a great advantage in bringing health professionals together in situations that require a multidisciplinary approach nurse, dietitian, psychologist, and others. (N5).

For many, these opportunities were also linked to professional development, requiring new competencies in digital communication, ethical documentation, and patient data management. Participants recognized that education and institutional support would be essential to fully integrate these emerging roles.

#### Motivation and new possibilities for younger nurses

A distinct generational perspective emerged among younger nurses, who expressed enthusiasm and curiosity about using technology to extend care beyond the bedside. They viewed remote healthcare as innovative, flexible, and aligned with their digital literacy and career aspirations.

As a young nurse, I find these applications exciting. I could track how my ICU patient manages care at home. (N1).

I’ve used apps and online platforms all my life, so it lets me combine patient care with new digital tools. (N22).

This subtheme reveals that for younger nurses, telehealth represents not only a new work modality but also a motivational factor an avenue to stay engaged in the profession and apply their digital skills meaningfully. Participants also pointed out that telehealth could offer flexible work arrangements, which might help address burnout and retain early-career nurses in clinical practice.

Remote healthcare makes me feel that I could continue to work even in situations where I might otherwise need to leave bedside care, such as family responsibilities or relocation. (N7).

The enthusiasm among younger nurses contrasted with a more cautious stance observed in senior participants, suggesting a potential generational shift in how digital health is perceived within nursing. As one participant noted, digital systems “excite the new generation and keep them motivated,” underscoring the evolving dynamics of workforce engagement in nursing’s digital era.

### Theme 3: Technological and organisational requirements

While nurses recognized the value of remote healthcare in improving patient care and professional roles, they also emphasized that its success depends on adequate technological infrastructure, effective coordination, and organizational readiness. Two interrelated subthemes were identified:

1. *Infrastructure and Devices*,
2. *Staffing and Workflow Coordination.*

#### Infrastructure and devices

Participants repeatedly noted that reliable technological infrastructure and accessible digital tools are essential prerequisites for remote healthcare. Inadequate internet connectivity, limited access to devices, and insufficient technical support were cited as major barriers to implementation.

Our country is not ready in terms of infrastructure. System crashes and internet access are major issues. (N21).

Devices such as imaging cameras, computers, thermometers, blood pressure monitors, and blood glucose meters should be available to monitor patients remotely. For the process to run smoothly, technological equipment must be very advanced. (N7).

Several nurses expressed concern that patients living in rural areas or low-resource settings may face difficulties due to limited internet access or outdated technology, potentially creating inequities in access to digital care. Others noted that even when devices were available, maintenance, calibration, and integration with existing hospital systems posed additional challenges.

Applicability would be limited in patients with technological infrastructure deficiencies or no internet access. (N14).

This subtheme highlights how technological readiness is not only a technical issue but also a social determinant of equitable care delivery, requiring national-level planning and investment. Nurses also acknowledged that they themselves need training to feel confident using these technologies safely and effectively.

#### Staffing and workflow coordination

Participants underscored that the organization and coordination of care teams are as crucial as the technology itself. They emphasized that remote healthcare must operate within a well-defined system that outlines scheduling, role distribution, and patient provider communication channels.

There must be a clear work schedule; who the patient contacts outside office hours must be defined. (N11).

Remote healthcare services should be well organized, including who will provide them and at what times. How will patients reach their nurse or doctor outside working hours? (N21).

Many nurses voiced concern about staffing shortages, noting that current workforce levels are already strained by face-to-face responsibilities. They feared that remote healthcare could unintentionally increase workloads unless new staffing models or dedicated teams are introduced.

We can’t provide remote healthcare services under our current circumstances. Both the technological infrastructure and the number of nurses are insufficient. Such an application would increase our workload. (N9).

The current healthcare workforce is designed for in-person care. To implement remote services safely, the workforce must be well-planned and adequate. (N20).

Participants recommended the development of structured appointment systems, dedicated telehealth shifts, and administrative support to prevent overburdening clinical nurses. The subtheme reveals that organizational planning and human resource management are perceived as decisive factors in sustaining remote healthcare.

This finding resonates with prior research emphasizing that successful telehealth integration requires both technological and organizational maturity, ensuring that digital solutions complement rather than compete with existing nursing workflows.

### Theme 4: Legal, ethical, and privacy concerns

Nurses consistently emphasized that successful remote healthcare delivery requires robust legal frameworks and ethical safeguards to protect both patients and professionals. They expressed concerns about data security, patient confidentiality, documentation practices, and professional accountability. Two subthemes were identified:

1. *Data Protection and Confidentiality. *
2. *Responsibility and Accountability.*

#### Data protection and confidentiality

Participants viewed data privacy and security as major challenges in remote healthcare implementation. They highlighted the need for clear national regulations that define data ownership, storage, and sharing processes. Several nurses noted that patients may feel uncomfortable sharing personal information digitally, particularly when care is provided by someone other than their primary nurse or physician.

National policies and laws must be developed before implementation. (N2).

This can create problems with data privacy. Patients may be uncomfortable with their data being shared digitally. If they don’t receive care from their own nurse or doctor, they may become anxious and experience difficulties. (N5).

Many participants questioned how long digital records would be stored, who would have access, and whether systems could ensure privacy across platforms. They worried that both patients and nurses could be exposed to unintended breaches during online consultations.

How much of the patient’s data will be in the system and for how long? How will this data be stored and accessed, and how will data security be ensured? (N16).

Remote care sounds good, but what if someone else is listening on the patient’s or professional’s side? It carries risks regarding data privacy and security. (N6).

Nurses also stressed that informed consent procedures for remote healthcare must be explicit, specifying how patient data are recorded, stored, and used.

Procedures regarding legal processes, data sharing, and confidentiality must be well prepared. Patient consent must be obtained before the procedure; how consent will be obtained and how records will be kept must all be clear and explicit. (N21).

This subtheme reveals a strong awareness among nurses of ethical responsibility in digital care. They recognized that, while technology enables access, it also demands heightened professional vigilance in protecting patient rights.

#### Responsibility and accountability

Nurses expressed uncertainty about who would bear legal responsibility in the event of errors or adverse outcomes resulting from remote consultations. They identified risks related to inconsistent documentation, ambiguous professional boundaries, and unclear lines of accountability within multidisciplinary teams.

“When a patient receives remote care from multiple healthcare professionals, everyone should convey the same message. What happens when the nurse’s and doctor’s recommendations to the patient vary? (N5).

Without proper documentation, the patient or a family member could claim something that never happened. Without secure records, who will be held accountable? Therefore, I consider this practice risky. (N3).

Several nurses also noted that documentation procedures for remote interactions must be standardized to ensure traceability. Lack of consistent documentation, they warned, could expose nurses to professional liability.

If not well organized, medical errors can increase. If the nurse doesn’t know the patient beforehand, it can be a major problem. Therefore, the system should pair the patient with healthcare professionals who already know them. (N20).

The service provided to the patient must be recorded. Otherwise, who will be responsible if complications arise during the service? Records of such situations constitute evidence if false claims are made. (N13).

This subtheme reflects nurses’ desire for legal clarity and institutional protection. They supported the establishment of clear national and organizational policies that define professional boundaries, liability distribution, and ethical documentation standards. Their comments reveal that maintaining trust, transparency, and accountability in remote healthcare is perceived as both a moral and practical necessity.

### Theme 5: Barriers related to users

Nurses frequently discussed user-related barriers that may limit the effectiveness of remote healthcare services. These barriers were related to digital literacy, motivation, and patient characteristics, as well as concerns about loss of therapeutic interaction between nurses and patients. Two interrelated subthemes were identified:

1. *Patient-Related Barriers,*
2. *Loss of Human Connection.*

#### Patient-related barriers

Participants emphasized that many patients, particularly older adults and those with low education levels, may lack the technological competence required to engage in remote monitoring systems. Difficulties in using mobile devices, poor internet access, or lack of awareness about digital applications were commonly reported.

For remote healthcare to work, patients need to know how to use the system. Some don’t even know how to answer a video call. (N10).

Patients with low education levels or older adults may not have the skills to use mobile phones or tablets for healthcare purposes. (N16).

Nurses also highlighted the challenge of maintaining patient motivation over time. They observed that while patients often engage enthusiastically at the beginning, adherence to self-monitoring tends to decline without continuous support or follow-up.

At first, patients are interested, but after a while, they lose motivation. They need regular encouragement and reminders to keep using the system. (N17).

In addition, several nurses noted that patients with cognitive impairments, mental health conditions, or language barriers may find it difficult to interact with digital systems safely or accurately. These limitations, they warned, could lead to incomplete assessments or missed warning signs.

It’s very difficult to follow up with patients who have hearing problems or Alzheimer’s disease. They can’t manage the system or respond correctly. (N8).

Collectively, these insights reveal that remote healthcare requires not only technology but also ongoing education, adaptation, and individualized support for patients and families.

#### Loss of human connection

Nurses expressed concern that remote healthcare might reduce interpersonal communication and weaken the emotional bond between nurses and patients an essential element of care. They described face-to-face interaction as fundamental to assessing patient needs, emotions, and nonverbal cues that are often missed during virtual encounters.

Some patients might not want to talk on camera. Without seeing their facial expressions or body language, it’s hard to understand their real condition. (N3).

Nursing is based on communication and human connection. You can’t replace the feeling of sitting beside a patient or touching their hand with a screen. (N11).

Participants feared that overreliance on digital tools could depersonalize care and diminish the therapeutic relationship that helps build trust and adherence. At the same time, several nurses acknowledged that blended approaches combining in-person and digital interactions could preserve human contact while leveraging the advantages of technology.

Some services can be provided remotely, but others will always require face-to-face contact. Remote care can support us, not replace us. (N20).

This subtheme highlights nurses’ deep awareness of the relational dimension of care and the importance of maintaining empathy, presence, and trust in an increasingly digital environment. Their reflections underscore that successful remote healthcare models must balance technological efficiency with the core values of compassionate nursing practice.

### Theme 6: Clinical appropriateness and boundaries

Nurses emphasized that remote healthcare services are not universally applicable to all patient populations or clinical conditions. Instead, they viewed such systems as suitable for specific groups and contexts, depending on patients’ physical condition, disease type, and level of self-care ability. Two subthemes were identified:

1. *Appropriateness of Patient and Condition Characteristics.*
2. *Appropriateness of Service Type.*

#### Appropriateness of patient and condition characteristics

Participants agreed that remote healthcare could be particularly effective for patients with chronic illnesses who require regular monitoring and guidance but are clinically stable. These include individuals with diabetes, hypertension, heart failure, respiratory diseases, or long-term rehabilitation needs.

It can be applied to patients with chronic diseases such as diabetes and hypertension who need close monitoring but are stable enough to be followed at home. (N19).

For post-surgery patients or those needing wound follow-up, remote healthcare can prevent unnecessary hospital visits while allowing us to check their healing process. (N14).

However, several nurses noted that remote healthcare is not appropriate for acute or unstable patients, or for those requiring physical examination and immediate intervention.

This method is not suitable for patients whose condition can change suddenly, like ICU patients. Some cases require direct observation and prompt intervention. (N1).

Participants also mentioned that the patient’s cognitive status, family support, and communication ability significantly influence suitability.

It can be applied to patients who are conscious, cooperative, and have a family member who can help when needed. (N4).

This subtheme underscores nurses’ capacity to differentiate between clinical conditions, advocating for judicious patient selection to maximize safety and effectiveness.

#### Appropriateness of service type

Nurses also reflected on which healthcare services are most feasible for remote delivery. They identified education, counseling, chronic follow-up, and medication adherence support as the most suitable. These activities align with nursing roles focused on health promotion and prevention rather than acute procedural care.

Remote healthcare could be very effective for patient education, counseling, or medication follow-up. But for treatments or emergencies, it’s not practical. (N9).

Services like nutrition counseling, mental health support, or chronic disease control can easily be provided remotely. (N2).

At the same time, participants emphasized that some services such as invasive procedures, physical examinations, and hands-on care must remain face-to-face. They advocated for a hybrid model combining digital and in-person approaches, depending on patient need and service type.

There are cases where we can’t touch or observe the patient closely. In those cases, remote healthcare should support, not replace, direct care. (N13).

This subtheme reveals nurses’ nuanced understanding of remote healthcare’s limits and possibilities. Rather than rejecting or idealizing the model, participants proposed a balanced, evidence-based approach that integrates digital and traditional care methods.

## Discussion

This study explored nurses’ perspectives on remote healthcare delivery, revealing a complex but optimistic view of its potential for improving patient outcomes, professional development, and system efficiency. Overall, nurses perceived remote healthcare as a transformative yet context-dependent model of care that can enhance continuity, accessibility, and patient engagement when supported by appropriate infrastructure, education, and ethical frameworks. The six themes identified covering benefits, professional opportunities, technological and organizational requirements, ethical and legal concerns, user-related barriers, and specific suitability illustrate the multifaceted realities of implementing remote care in everyday nursing practice.

The findings align with growing international evidence emphasizing that nurses are central actors in digital health transformation, serving as mediators between technology, patients, and systems. Studies conducted in diverse contexts have shown that nurse-led telehealth interventions improve chronic disease outcomes, enhance patient self-management, and promote more efficient use of healthcare resources [5, 6]. Similar to prior research, the nurses in this study viewed remote healthcare as a natural extension of nursing practice, particularly for chronic disease management, health education, and patient follow-up. However, they also highlighted the need for structured training, role clarity, and organizational support, underscoring that technology alone cannot guarantee quality or safety in care delivery.

These findings extend previous research by providing insight from Turkiye a context in which telehealth adoption is expanding yet remains uneven across institutions and regions [24]. Nurses described systems that continue to operate within predominantly physician-centered structures, where nursing documentation is often fragmented or paper-based. Such limitations constrain the visibility of nursing contributions within digital health environments and may help explain why issues related to role clarity, professional visibility, and cautious adaptation featured so prominently in our findings. In environments where digital systems are only partially integrated, remote healthcare is not experienced as a seamless innovation but rather as an evolving practice that requires ongoing negotiation.

Within the Turkish healthcare context, barriers such as institutional variability in digital infrastructure, limited technological resources, inadequate institutional protocols, insufficient integration of hospital information systems, lack of training and digital competencies, the absence of standardized national nursing documentation frameworks, unclear nursing roles and responsibilities in remote care processes, and reliance on hybrid paper-electronic documentation models all contribute to this fragmentation.These factors do more than slow implementation; they influence how nursing work is recorded, recognized, and legitimized within digital health environments.

This reflects a broader global concern: while digital transformation is advancing rapidly, nursing systems frequently lag in developing nurse-centered documentation standards and interoperable data structures that can capture the full scope of nursing care [14, 15]. Although similar tensions have been reported internationally, particularly where digital transformation advances faster than the development of nurse-centered documentation standards and interoperable data structures the uneven pace of integration in Turkiye may render these professional challenges more visible. Addressing these structural gaps is essential not only for efficiency but also for professional recognition and accountability within remote healthcare systems and for supporting the sustainable integration of remote healthcare into everyday nursing practice.

Another important contribution of this study is its illustration of nurses’ ethical and relational consciousness in the digital era. Participants emphasized that legal regulation, patient consent, and data security are indispensable components of remote healthcare implementation. This echoes previous studies indicating that nurses’ ethical awareness directly influences their trust in technology and their willingness to integrate digital tools into care [ 23, 4]. Moreover, the nurses’ concern about preserving human connection in digital care reflects a core nursing value caring as presence which remains essential even as care modalities evolve. Their reflections suggest that empathy, trust, and touch are not obsolete but must be reinterpreted within new technological contexts.

From an educational perspective, these findings point to the need for more deliberate integration of telehealth competencies and ethical digital practice with in both undergraduate and continuing nursing education. As participants emphasized, the ability to communicate effectively online, ensure data security, and maintain professional boundaries no longer optional skills but emerging core competencies for contemporary nursing practice. Preparing nurses for remote care therefore requires more than technical instruction; it involves cultivating digital communication judgment, ethical awareness, and confidence in navigating evolving technological systems. Educational programs may need to address generational differences by recognizing younger nurses’ relative digital fluency while also providing structured support for more experienced nurses adapting to new care modalities. Simulation-based telehealth training, mentorship models, and practice-integrated learning opportunities could facilitate this transition across experience levels. These programmes should focus on digital communication skills, data literacy, ethical decision-making, virtual patient assessment and interprofessional collaboration within digital environments [25]. This will ensure that all nurses are equipped to navigate the evolving demands of telehealth practice.

Finally, the study suggests that the success of remote healthcare depends as much on organizational and policy readiness as individual competence. Participants’ accounts indicate that technological infrastructure, workforce planning, and clear legal frameworks cannot evolve in isolation; they must develop in parallel if remote care is to be sustainable. In the absence of such alignment, nurses may encounter increased workload, ethical uncertainty, and professional vulnerability. Sustainable implementation therefore requires a whole-system approach aligning technology, governance, education, and nursing leadership. Within this framework, clarifying nurses’ roles and establishing consistent protocols appear particularly critical. Structured training and ongoing professional development can support nurses in using telemonitoring systems, electronic health records, and secure data management tools with confidence.

At the practice level, integrating face-to-face and remote workflows in a coordinated manner may help prevent fragmentation of care. Strengthening multidisciplinary communication across nurses, physicians, technical staff, and other healthcare professionals is equally important. Ongoing monitoring of service quality and patient safety, accompanied by feedback mechanisms, can further support sustainable implementation. Together, these measures support a more coherent and professionally grounded integration of nursing within evolving remote healthcare systems [25, 26].

### Limitation

Several limitations of this study should be considered when interpreting the findings.

First, the sample included nurses from selected healthcare institutions in Turkiye and therefore cannot fully represent the diversity of organisational structures and regional conditions across the country. Remote healthcare practices in Turkiye vary considerably depending on institutional readiness, technological infrastructure, and managerial support. As a result, the experiences described here reflect specific organisational contexts rather than a uniform national model of remote care delivery.

Second, the study relied on self-reported experiences gathered through qualitative interviews. The findings therefore represent how nurses perceive and make sense of remote healthcare rather than directly observed clinical behaviours. These perceptions are inevitably shaped by individual factors such as digital literacy, prior telehealth exposure, professional identity, and workplace culture. Consequently, interpretations related to effectiveness or quality should be understood as experiential and contextual rather than outcome-based.

Third, although participants were recruited from both hospital and community settings, the majority were clinical nurses. This imbalance may have contributed to a stronger emphasis on hospital-based workflows and constraints within the findings. Experiences specific to community-based remote care may therefore be underrepresented.

Fourth, the study focused exclusively on nursing perspectives. Remote healthcare delivery is inherently interdisciplinary, involving collaboration with physicians, information technology professionals, administrators, and patients. The absence of these viewpoints limits system-level interpretation and means that the findings should be understood primarily as representing the nursing dimension of remote healthcare implementation.

Fifth, geographical and structural disparities within Türkiye including rural urban differences, regional variation in digital infrastructure, and uneven levels of digital maturity were not analysed in depth. These contextual factors likely influence both access to and adaptation of remote healthcare practices. As digital transformation continues to progress at different speeds across regions, nurses’ experiences may also vary accordingly.

Importantly, remote healthcare implementation in Türkiye remains in a dynamic phase of development. The findings reflect a particular moment within this ongoing transformation, characterised by partial institutional integration and evolving regulatory and reimbursement frameworks. For this reason, the recommendations offered in this study should be interpreted as context-sensitive rather than universally prescriptive.

While these limitations constrain the scope of generalisation, they also underscore the importance of situating telehealth research within specific health system contexts. Countries undergoing similar stages of digital transformation may recognise comparable patterns, whereas systems with more established telehealth infrastructures may demonstrate different professional experiences.

## Conclusion

This study offers an in-depth understanding of nurses’ perspectives on remote healthcare delivery, showing that nurses perceive digital care as a valuable yet context-dependent model of practice. Remote healthcare was viewed as a means to strengthen continuity, accessibility, and chronic disease management, while also expanding the scope and visibility of nursing roles. At the same time, participants identified essential prerequisites for safe implementation, including adequate infrastructure, clearly defined organizational processes, and robust legal and ethical safeguards.

Nurses expressed both optimism and caution: optimism regarding the potential of digital tools to support holistic and patient-centered care, and caution concerning workload, accountability, and the preservation of human connection. Their reflections reinforce the view that digital health should complement rather than replace face-to-face care, sustaining empathy and relational presence as foundational nursing values.

From an educational perspective, these findings highlight the need to integrate telehealth competencies and digital ethics into undergraduate and continuing nursing education. Preparing nurses for responsible remote care requires structured training in digital communication, data security, documentation practices, and interprofessional coordination. At the system level, healthcare leaders and policy-makers should recognize nurses not merely as users of technology but as active contributors to the design and governance of remote care systems.

Collectively, the findings underscore the importance of embedding nurses’ experiential knowledge into implementation strategies. As central coordinators of care across digital and in-person contexts, nurses play a pivotal role in ensuring patient safety, continuity, and equity. Sustaining remote healthcare in the long term therefore depends on aligning technological development with professional accountability, organizational clarity, and the relational foundations of nursing practice.

### Recommendations or implications for practice and/or further research

Integrating telehealth competencies into undergraduate and continuing nursing education emerges as a key priority for preparing nurses to work effectively in digital care environments. Based on participants’ accounts, educational initiatives should emphasise digital communication skills, ethical decision-making, documentation standards, and data security, while being supported by institutional investment in technological infrastructure and workforce development. At the policy level, sustainable implementation of remote healthcare will depend on strengthened digital infrastructure, improved interoperability between systems, and clear legal frameworks that define professional roles and accountability. Active engagement of nursing leadership in digital health governance is essential to ensure that remote care models reflect nursing practice realities.

Future research should extend beyond single-setting analyses to include comparative studies across primary, secondary, and tertiary care contexts. Interdisciplinary investigations involving physicians and allied health professionals would also provide a more comprehensive understanding of system-level implementation. Further work is needed to examine geographical variability, particularly in rural and resource-limited settings, to inform the development of equitable and context-responsive remote healthcare models.

## Data Availability

The data that support the findings of this study are available from the corresponding author upon reasonable request.
